# Early Plasmapheresis Among Patients With Hypertriglyceridemia–Associated Acute Pancreatitis

**DOI:** 10.1001/jamanetworkopen.2023.20802

**Published:** 2023-06-28

**Authors:** Longxiang Cao, Yingjie Chen, Siyao Liu, Wei Huang, Dong Wu, Donghuang Hong, Zuozheng Wang, Yi Sun, Kaixiu Qin, Feng Guo, Cuizhu Luo, Qinghai Jiao, Xiang Luo, Jing Zhou, Gang Li, Bo Ye, Tao Chen, Man Liu, Wenjian Mao, Lanting Wang, Shuai Li, John A. Windsor, Yuxiu Liu, Lu Ke, Zhihui Tong, Weiqin Li

**Affiliations:** 1 Department of Critical Care Medicine, Jinling Hospital, Medical School of Nanjing University, Nanjing, China; 2 National Institute of Healthcare Data Science, Nanjing University, Nanjing, China; 3 Department of Critical Care Medicine, Hospital of Chengdu University of Traditional Chinese Medicine, Chengdu, China; 4 Department of Critical Care Medicine, Jinjiang Hospital of Traditional Chinese Medicine, Quanzhou, China; 5 Department of Emergency Medicine, The First Affiliated Hospital of Xiamen University, Xiamen, China; 6 West China Center of Excellence for Pancreatitis, Institute of Integrated Traditional Chinese and Western Medicine, West China-Liverpool Biomedical Research Center, West China Hospital, Sichuan University, Chengdu, China; 7 Department of Gastroenterology, State Key Laboratory of Complex Severe and Rare Diseases, Peking Union Medical College Hospital, Chinese Academy of Medical Sciences and Peking Union Medical College, Beijing, China; 8 Department of Critical Care Medicine, Fujian Provincial Hospital, Fuzhou, China; 9 Department of Hepatobiliary Surgery, General Hospital of Ningxia Medical University, Yinchuan, China; 10 The Fourth Department of the Digestive Disease Center, Suining Central Hospital, Suining, China; 11 Department of Emergency Medicine, The Second Affiliated Hospital of Chongqing Medical University, Chongqing, China; 12 Department of Intensive Care Unit, Sir Run Run Shaw Hospital of Zhejiang University School of Medicine, Hangzhou, China; 13 Department of Critical Care Medicine, Pingxiang People's Hospital, Pingxiang, China; 14 Department of Critical Care Medicine, The First Hospital of Handan, Handan, China; 15 Department of Critical Care Medicine, Longyan First Affiliated Hospital of Fujian Medical University, Longyan, China; 16 Department of Critical Care Medicine, Jinling Hospital, Nanjing Medical University, Nanjing, China; 17 Department of Public Health, Policy and Systems, Institute of Population Health, The University of Liverpool, Liverpool, United Kingdom; 18 Surgical and Translational Research Center, Faculty of Medical and Health Sciences, University of Auckland, Auckland, New Zealand; 19 Department of Biostatistics, School of Public Health, Southern Medical University, Guangzhou, China

## Abstract

**Importance:**

The incidence of hypertriglyceridemia–associated acute pancreatitis (HTG-AP) is increasing. Plasmapheresis is theoretically effective in removing triglyceride from plasma, but whether it confers clinical benefits is unclear.

**Objective:**

To assess the association between plasmapheresis and the incidence and duration of organ failure among patients with HTG-AP.

**Design, Setting, and Participants:**

This is an a priori analysis of data from a multicenter, prospective cohort study with patients enrolled from 28 sites across China. Patients with HTG-AP were admitted within 72 hours from the disease onset. The first patient was enrolled on November 7th, 2020, and the last on November 30th, 2021. The follow-up of the 300th patient was completed on January 30th, 2022. Data were analyzed from April to May 2022.

**Exposures:**

Receiving plasmapheresis. The choice of triglyceride-lowering therapies was at the discretion of the treating physicians.

**Main Outcomes and Measures:**

The primary outcome was organ failure–free days to 14 days of enrollment. Secondary outcomes included other measures for organ failure, intensive care unit (ICU) admission, duration of ICU and hospital stays, incidence of infected pancreatic necrosis, and 60-day mortality. Propensity score matching (PSM) and inverse probability of treatment weighting (IPTW) analyses were used to control potential confounders.

**Results:**

Overall, 267 patients with HTG-AP were enrolled (185 [69.3%] were male; median [IQR] age, 37 [31-43] years), among whom 211 underwent conventional medical treatment and 56 underwent plasmapheresis. PSM created 47 pairs of patients with balanced baseline characteristics. In the matched cohort, no difference was detected concerning organ failure–free days between patients undergoing plasmapheresis or not (median [IQR], 12.0 [8.0-14.0] vs 13.0 [8.0-14.0]; *P* = .94). Moreover, more patients in the plasmapheresis group required ICU admission (44 [93.6%] vs 24 [51.1%]; *P* < .001). The IPTW results conformed to the results from the PSM analysis.

**Conclusions and Relevance:**

In this large multicenter cohort study of patients with HTG-AP, plasmapheresis was commonly used to lower plasma triglyceride. However, after adjusting for confounders, plasmapheresis was not associated with the incidence and duration of organ failure, but with increased ICU requirements.

## Key Points

**Question:**

Plasmapheresis is theoretically effective in removing triglyceride from plasma, but is it associated with clinical outcomes in patients with hypertriglyceridemia-associated acute pancreatitis (HTG-AP)?

**Findings:**

In this multicenter cohort study involving 267 patients with HTG-AP, plasmapheresis was not associated with the incidence and duration of organ failure, but with a greater need for intensive care unit admission.

**Meaning:**

These findings suggest plasmapheresis may not be used in the management of HTG-AP because of the cost and potential complications and because it may not confer any clinical benefit.

## Teaser

This cohort study evaluates the association between plasmapheresis and the incidence and duration of organ failure among patients with hypertriglyceridemia–associated acute pancreatitis.

## Introduction

Acute pancreatitis (AP) is a common gastrointestinal disease with multiple causes, including gallstones, alcohol, and hypertriglyceridemia (HTG).^1^ In recent years, HTG has become the third most common cause globally, accounting for 4% to 10% of all AP cases.^2^ In China, elevated HTG has become the second leading cause of AP in recent years.^3^ Although the pathophysiology of HTG–associated AP (HTG-AP) is not fully understood, it has been shown that increased plasma triglyceride levels may be associated with worse clinical outcomes.^4^

On that basis, there have been a number of treatment strategies introduced to reduce plasma triglyceride levels, including noninvasive measures such as fasting, insulin, and heparin and invasive blood purification techniques.^2^ Plasmapheresis, which replaces plasma with other fluids such as fresh plasma or albumin, has been widely studied, but the evidence is contradictory.^5,6,7^ Not only would plasma triglyceride be removed by plasmapheresis, but there is the expectation that chylomicrons and inflammatory cytokines would also be efficiently removed.^8^

Most of the studies investigating the impact of plasmapheresis on the outcome from HTG-AP were small, retrospective, and often included mild cases.^6,9,10,11,12,13,14,15,16,17^ The only randomized controlled trial showed no effect of plasmapheresis on plasma triglyceride levels and clinical outcomes.^18^ Despite this evidence base, the American Society for Apheresis guidelines recommended plasmapheresis for severe HTG-AP and to prevent relapse,^8^ although they acknowledged that the evidence was either low or very low quality. International guidelines for the management of AP did not give any recommendations regarding specific triglyceride-lowering therapy due to the lack of solid evidence.^19,20,21,22^ In this study, we aimed to assess the association between plasmapheresis and the incidence and duration of organ failure in patients with HTG-AP using data from a multicenter, prospective observational study.

## Methods

### Study Design

This study was an a priori analysis of data collected for the PERFORM study, which was registered in the Chinese Clinical Trials Registry. The PERFORM study^23^ was a multicenter, prospective cohort study collecting clinical characteristics, treatments, and outcomes of patients with HTG-AP. It was designed and conducted by the Chinese Acute Pancreatitis Clinical Trials Group (CAPCTG) and approved by the hospital ethics committees of all the participating hospitals. Written informed consent was obtained from each participant or their next of kin. The full protocol of the PERFORM study and the analysis plan of this study were published previously.^23^ This report follows the Strengthening the Reporting of Observational Studies in Epidemiology (STROBE) reporting guideline for observational studies.^24^

### Patient Characteristics

All patients admitted with acute pancreatitis to the participating hospitals were considered for eligibility for the PERFORM study. The inclusion criteria were adult patients (18-70 years), admitted within 72 hours from the onset of pain, triglyceride level greater than 11.3 mmol/L when enrolled (to convert to millimoles per liter, multiply by 0.0113), and the presence of at least 1 of the worrisome features, which were defined and described in detail by Gelrud et al^25^ on UpToDate. The list of worrisome features can be found in the published protocol.^23^ The exclusion criteria included failure to obtain informed consent, pregnant or lactating women, and patients who were expected to die within 48 hours after enrollment. In this analysis, we additionally excluded patients who underwent any type of blood purification other than plasmapheresis for triglyceride-lowering therapy.

### Patient Treatment and Triglyceride-Lowering Therapies

All the patients received standardized treatment according to guidelines, including intravenous fluid, early enteral nutrition, and delayed intervention for local complications.^19^ For triglyceride-lowering therapy, patients who underwent at least 1 plasmapheresis session were assigned to the plasmapheresis group, and those who did not were assigned to the conventional group. The choice of triglyceride-lowering therapies was at the discretion of the treating physicians. Plasmapheresis included therapeutic plasma exchange (TPE) and double filtration plasmapheresis (DFPP).

### Study Outcomes and Definitions

The primary outcome was organ failure–free days (OFFD) to 14 days of enrollment, defined as the number of days alive without failure of respiration, kidney, or cardiovascular organ systems.^26^ An individual Sequential Organ Failure Assessment (SOFA) score of 2 or more was defined as organ failure. Only the final period of OFFD was counted. Patients discharged from the hospital before 14 days were considered alive and free from organ failure since the day of discharge. Patients who died before day 14 were assigned zero OFFD.

Secondary outcomes included new-onset organ failure to day 14, SOFA_rank_, and change in SOFA_max_, intensive care unit (ICU)–free days to day 14, ICU need, hospital-free days to day 60, mortality, and incidence of infected pancreatic necrosis by day 60 of enrollment and laboratory results including triglyceride level within 3 days after enrollment and C-reactive protein level within 7 days after enrollment. New-onset organ failure was defined as organ failure that is not present in the first 24 hours after enrollment. SOFA_rank_ was a ranking parameter according to the cumulative daily change in SOFA score from day 1 to day 14. For each patient, it was calculated as a sum of the daily change in SOFA score (defined as the daily total SOFA score minus the baseline SOFA score) over the first 14 study days.^27^ Discharge was counted (from the day of discharge forward) as a score of 0 minus baseline score, and death was counted (from the day of death forward) as a maximum score of 24 minus baseline score. The resulting cumulative daily change score was used to rank participants from fast organ failure resolution (lowest scores) to worsening organ failure and death (highest scores). Change in SOFA_max_ was defined as the maximum SOFA score within 14 days minus the baseline SOFA score.^28^ The definition of other secondary outcomes can be found in the published protocol.^23^

### Data Collection

In this study, all data were extracted from the electronic database (Unimed Scientific Inc) of the PERFORM study (phase 1, including 300 participants), including deidentified data on demographic characteristics, clinical data concerning the daily treatment and laboratory results, and the follow-up data on day 60 of enrollment. All the data were collected and stored in a secure web-based database, and the coordinating center of CAPCTG is responsible for the safety and integrity of the collected data. The follow-up on day 60 was implemented through telephone. More details regarding data collection can be found in the published protocol and the website of CAPCTG.^23^

### Statistical Analysis

The normality for continuous variables was determined by the Shapiro-Wilk test. Continuous normally distributed data were reported as mean (SD). Skewed continuous data were reported as median (IQR). Categorical data were summarized by counts and percentages. The intergroup difference was compared by *t* test or Wilcoxon rank-sum test for continuous variables depending on their normality and the χ^2^ test or Fisher exact test for categorical data.

Propensity score matching (PSM) analysis was used to control potential confounders. Patients who received plasmapheresis were matched 1:1 with patients who received conventional treatment using their propensity score. We followed 3 rules to choose the variables for PSM: (1) potential baseline differences between groups with a *P* value less than .10; (2) potentially relevant variables according to previous studies and clinical considerations; and (3) missing data less than 10%. Collinearity was additionally tested to ensure the independence of each variable. As a result, age,^29,30^ sex,^4,30^ body mass index^30^ (BMI), baseline triglyceride level,^30^ baseline Acute Physiology and Chronic Health Evaluation II (APACHE II) score,^31,32,33^ and the baseline SOFA score^4^ were involved. Genetic matching with a caliper width of 0.3 was used in the PSM. Standardized mean difference was used to assess the balance of baseline covariates between treatment groups in the matched sample with that in the unmatched sample. A standardized mean difference of more than 0.1 and a 2-sided *P* value of less than .05 indicated a significant imbalance in the baseline covariate.

For the matched pairs, the difference in binomial outcomes between groups was assessed with risk difference and 95% CIs. The differences in continuous outcomes were assessed with a median difference and 95% CIs calculated with the Hodges-Lehmann estimation of location shift. The *P* value was calculated with the Wilcoxon signed-rank test and McNemar test for matched data.

Kaplan-Meier methods were used to show curves to organ failure resolution in the matched cohorts. A log-rank *P*-test stratified on the matched pairs was used to test the equality of the estimated survival curves. A Cox proportional hazards model that incorporated a robust sandwich-type variance estimator to account for the matched nature of the data was used to estimate cause-specific hazard ratios (HRs).^34,35^

To evaluate the robustness of our findings, we performed a sensitivity analysis using inverse probability of treatment weighting (IPTW) analysis with the same variables as PSM. Comparisons of differences between groups were performed using χ^2^ test for binary variables and Wilcoxon rank-sum test for continuous variables weighted by the inverse probability of treatment.

All analyses were performed using a uniform 2-sided test, with a significance level of .05, and presented with 2-sided 95% CIs. Analyses were performed using SAS software, version 9.4 (SAS Institute) and R software, version 4.1.1 (R Project for Statistical Computing). Data were analyzed from April to May 2022.

## Results

### Baseline Characteristics

**Figure 1.**
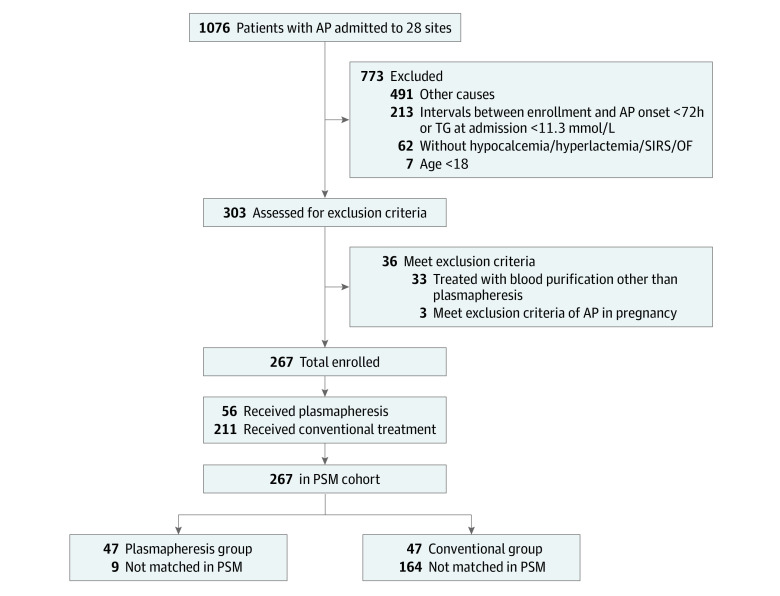
The Flow of Participants Through the Study To convert triglycerides (TG) to millimoles per liter, multiply by 0.0113. AP indicates acute pancreatitis; OF, organ failure; PSM, propensity score matching; SIRS, systemic inflammatory response syndrome.

The PERFORM registry achieved its phase 1 goal in January 2022, with 1076 patients from 28 sites assessed for eligibility. Among them, 300 were enrolled, and the 60-day follow-up was successfully implemented in all the patients (Figure 1). The first patient was enrolled on November 7, 2020, and the last on November 30, 2021. The follow-up of the 300th patient was completed on January 30, 2022. After excluding 33 patients who underwent blood purification other than plasmapheresis, 267 patients were involved in this analysis.

Of the 267 patients, 56 received at least 1 plasmapheresis session, and 211 received conventional medical treatment. Patients in the plasmapheresis group had significantly higher APACHE II, SOFA, and systemic inflammatory response syndrome (SIRS) scores at enrollment than patients in the conventional group (Table 1). Baseline respiratory failure (24 [43%] vs 46 [22%]; *P* < .001, Fisher exact test) and cardiovascular failure (6 [11%] vs 1 [1%]; *P* < .001, Fisher exact test) were more frequent in the plasmapheresis group (Table 1).

**Table 1.** Baseline Characteristics of Plasmapheresis and Conventional Groups Before and After Propensity Score Matching

Characteristics	Participants before matching, No. (%)			Participants after matching, No. (%)		
	Plasmapheresis (n = 56)	Conventional (n = 211)	*P* value	Plasmapheresis (n = 47)	Conventional (n = 47)	*P* value
Age, median (IQR), y	36.5 (31.5-43.0)	37.0 (31.0-44.0)	.96	36.5 (31.8-43.0)	37.0 (28.5-43.0)	.76
Sex						
Male	33 (59)	152 (72)	.06	29 (62)	30 (64)	>.99
Female	23 (41)	59 (28)		18 (38)	17 (36)	
BMI, mean (SD)^a^	27.3 (4.1)	27.9 (4.4)	.70	28.0 (3.8)	26.8 (3.3)	.95
Smoking	26 (46)	73 (35)	.12	22 (47)	14 (30)	.14
Drinking	23 (41)	76 (36)	.54	21 (45)	17 (36)	.53
Acute pancreatitis history	28 (50)	120 (57)	.37	25 (53)	33 (70)	.14
Academic hospital	53 (95)	196 (93)	.77	44 (94)	40 (85)	.32
APACHE II score, median (IQR)	9.0 (6.0-14.0)	4.0 (2.0-7.0)	<.001	7.0 (4.0-12.0)	8.0 (5.8-11.0)	>.99
APACHE II ≥8	33 (59)	43 (20)	<.001	24 (51)	23 (49)	>.99
SOFA score, median (IQR)	2.0 (1.0-4.0)	1.0 (0.0-2.0)	<.001	2.0 (0.0-3.0)	2.0 (1.0-3.0)	.85
Respiratory failure	24 (43)	46 (22)	<.001	17 (36)	19 (40)	.83
Circulatory failure	6 (11)	1 (1)	<.001	1 (2)	0	>.99
Kidney failure	8 (14)	14 (7)	.12	5 (11)	5 (11)	>.99
SIRS score, median (IQR)	7.0 (5.0-10.0)	5.0 (3.0-7.0)	<.001	7.0 (5.0-7.8)	5.0 (4.0-9.5)	.71
CTSI score, median (IQR)	2.0 (2.0-4.0)	2.0 (1.0-4.0)	.54	2.0 (2.0-3.8)	4.00 (2.0-6.0)	.17
TG, median (IQR), mmol/L	23.8 (16.9-36.5)	20.9 (15.9-30.9)	.13	23.2 (16.9-29.6)	24.5 (17.8-38.5)	.88
CRP, median (IQR), mg/L	128.9 (26.4-193.6)	36.9 (8.4-125.4)	.009	145.4 (52.5-238.8)	164.9 (90.6-250.2)	.35
PCT, median (IQR), ug/L	1.9 (0.3-6.2)	0.3 (0.1-1.2)	.001	3.6 (0.1-9.0)	1.1 (0.3-2.9)	.83
Triglyceride-lowering therapies						
Insulin	46 (82)	177 (84)	.84	38 (81)	42 (89)	.39
Heparin	49 (8)	176 (83)	.54	42 (89)	36 (77)	.17
Fasting	56 (100)	211 (100)	NA	47 (100)	47 (100)	NA

Abbreviations: APACHE, Acute Physiology and Chronic Health Evaluation; BMI, body mass index; CRP, C-reactive protein; CTSI, computed tomography severity index; NA, not applicable; PCT, procalcitonin; SIRS, systemic inflammatory response syndrome; SOFA, Sequential Organ Failure Assessment; TG, triglyceride.

SI conversions: To convert CRP to milligrams per liter, multiply by 10; TG to milligrams per deciliter, divide by 0.0113.

^a^ Body mass index is calculated as weight in kilograms divided by height in meters squared.

### Propensity Score Matching

After PSM, 47 matched pairs were created. The imbalance in the baseline characteristics was significantly reduced after PSM (eFigure 1 in Supplement 1). The baseline characteristics of the entire study cohort and the PSM cohort are presented in Table 1. There was no significant difference between groups after PSM.

### Plasmapheresis

**Figure 2.**
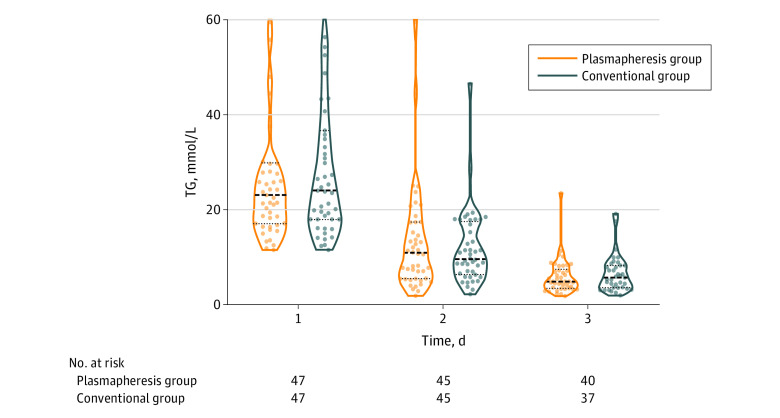
Daily Serum Triglyceride (TG) Levels in the Matched Cohort Violin plots show medians (thick dashed lines), interquartile ranges (thin dashed lines), and distribution of daily serum triglyceride levels among patients. To convert TG to milligrams per deciliter, divide by 0.0113.

The detailed characteristics of the plasmapheresis procedures are shown in eTable 1 in Supplement 1. In the unmatched cohort, 56 patients underwent plasmapheresis treatment, of whom 50 received TPE, and 6 received DFPP. For the timing of plasmapheresis, 40 patients underwent the first session on day 1 and 13 on day 2. Each session used a median (IQR) of 2000 (2000-2700) mL plasma and took a median (IQR) of 2.5 (2.0-3.0) hours. The median triglyceride levels of both groups from days 1 to 3 are shown in Figure 2, and there was no difference between groups for plasma triglyceride level on all the study days.

### Primary Outcome: OFFD

**Figure 3.**
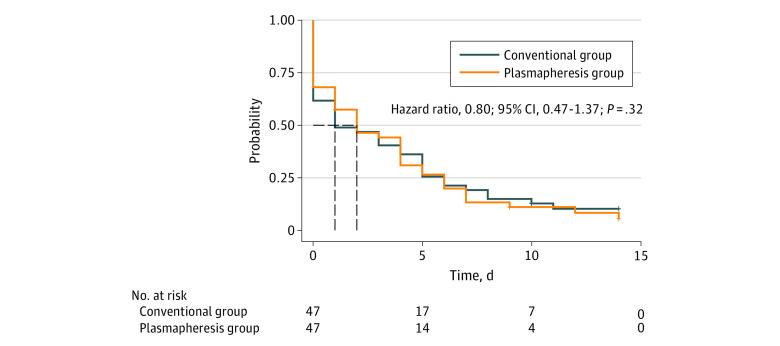
Time to Organ Failure Resolution by Day 14 in the Matched Cohort The Kaplan-Meier curves for the cumulative incidence of organ failure resolution from enrollment to day 14 in the matched cohort.

In the matched cohort, no difference was found in OFFD to day 14 between the plasmapheresis group and the conventional group (median [IQR], 12.0 [8.0 to 14.0] vs 13.0 [8.0 to 14.0]; median difference, 0.00; 95% CI, −1.00 to 1.00; *P* = .94). There was no difference in the probability of organ failure resolution (HR, 0.80; 95% CI, 0.47 to 1.37; log-rank *P* = .32) between the matched cohorts with Kaplan-Meier curves and Cox proportional hazards models (Figure 3).

### Secondary Outcomes

There was no difference between groups for SOFA_rank_, change in SOFA_max_, and new-onset organ failure (Table 2). The median C-reactive protein (CRP) levels and SOFA scores from days 1 to 7 are shown in eFigures 2 and 3 in Supplement 1, and there was no difference between groups for CRP levels and SOFA score on all the study days. However, patients in the plasmapheresis group, compared with the conventional group, had a greater need for ICU admission (44 [93.6%] vs 24 [51.1%], risk difference, 0.43; 95% CI, 0.27-0.58; *P* < .001). There were no differences in 60-day mortality, ICU-free days to day 14, or hospital-free days to day 60 between groups (Table 2).

**Table 2.** Outcomes of Plasmapheresis and Conventional Groups After Propensity Score Matching

Outcome	Patients, median (IQR)			*P* value
	Plasmapheresis (n = 47)	Conventional (n = 47)	Difference (95% CI)^a^	
Primary outcome				
OFFD to day 14	12.0 (8.0 to 14.0)	13.0 (8.0 to 14.0)	0.00 (−1.00 to 1.00)	.94
Secondary outcomes				
New-onset OF to day 14, No. (%)	20 (43)	14 (30)	0.13 (−0.06 to 0.32)	.31
SOFA_rank_	−11.0 (−28.0 to 0.0)	−12.0 (−26.0 to 1.0)	0.00 (−8.00 to 7.00)	.97
Change in SOFA_max_	1.0 (0.0 to 3.0)	1.0 (0.0 to 2.0)	0.00 (−1.00 to 1.00)	.51
ICU-free days to day 14	9.0 (6.0 to 11.0)	10.0 (6.0 to 14.0)	−1.00 (−3.00 to 1.00)	.20
Hospital-free days to day 60	46.0 (39.0 to 52.0)	52.0 (44.0 to 53.0)	−3.00 (−6.00 to 0.00)	.05
ICU need, No. (%)	44 (94)	24 (51)	0.43 (0.27 to 0.58)	<.001
60-day mortality, No. (%)	3 (6)	2 (4)	0.02 (−0.07 to 0.11)	>.99
IPN, No. (%)	6 (13)	3 (6)	0.06 (−0.05 to 0.18)	.51

Abbreviations: ICU, intensive care unit; IPN, infected pancreatic necrosis; OF, organ failure; OFFD, organ failure–free day; SOFA, Sequential Organ Failure Assessment.

^a^ Difference means the risk difference for binomial outcomes and the median difference for continuous outcomes calculated with a Hodges-Lehmann estimation of location shift between groups.

### Sensitivity Analysis

The PS distribution in the IPTW completely overlapped with the original cohort. The results showed no difference between groups in OFFD to day 14 (eTable 2 in Supplement 1). For secondary outcomes, the plasmapheresis group had fewer ICU-free days to day 14 (median [IQR], 10.0 [7.0-11.0] vs 14.00 [7.0-14.0]; *P* < .001) and greater need for ICU (264 [94%] vs 112 [36%], *P* < .001) compared with the conventional group. There was no difference in other secondary outcomes between groups.

## Discussion

In this large, prospective, multicenter cohort study involving patients with HTG-AP, no association between plasmapheresis and the incidence and duration of organ failure was observed. This finding held true after sensitivity analysis. Moreover, analyses of the secondary outcomes showed that plasmapheresis was not associated with an enhanced triglyceride-lowering effect compared with medical treatment, and it appeared to be associated with a greater need for admission to ICU.

A possible explanation for the findings is that plasmapheresis may not decrease triglyceride levels more efficiently than conventional medical therapy, as shown in our study and other observational studies conducted by Chen et al^36^ and Miyamoto et al.^37^ Recently, a randomized trial also demonstrated that, compared with insulin treatment, plasmapheresis did not result in more efficient triglyceride-lowering.^18^ Of note, the trial only involved patients presumed mild, and no data regarding organ function were shown, limiting its generalizability to patients with more severe conditions. In contrast, organ failure was present in 56% of patients (168 of 300) overall and in 69% of the matched cohort (65 of 94).

Although studies have investigated the clinical relevance of plasmapheresis in patients with HTG-AP, the results are discordant due to divergent study designs and quality. A study compared plasmapheresis combined with hemofiltration to hemofiltration alone and found that the combined treatment was associated with lower mortality and shorter hospital stay.^16^ Another study found DFPP was associated with reduced major complications in patients with HTG-AP with higher triglyceride levels.^9^ However, other studies found plasmapheresis was not associated with reduced mortality or length of hospital stay.^38,39,40,41,42,43^

Plasmapheresis has been used for decades in patients with HTG-AP because of its purported rapid triglyceride-lowering effects. Moreover, the Havel theory, the most widely accepted theory for the pathogenesis of HTG-AP, assumes that the lipid toxic effects of free fatty acids (FFA) to the pancreatic endothelium and acinar cells is the key mechanism.^44^ Singh et al^45^ also found that pancreatic enzymes can enter the surrounding visceral adipocytes in multiple ways, leading to the generation of excess nonesterified fatty acids. On that basis, it was thought that conventional plasma exchange, rather than DFPP (which did not remove FFA), may benefit patients with HTG-AP by removing FFA from the patient's plasma.^46^ However, plasma FFA levels were similar between patients undergoing plasmapheresis and those undergoing insulin therapy in a recent randomized trial.^18^ Due to the challenges in maintaining laboratory control over multiple sites, we did not measure FFA levels in this study.

In this study, the use of plasmapheresis was associated with a greater need for ICU admission. Technically, plasmapheresis is an invasive treatment that requires central venous access, specific devices, and rigorous monitoring for coagulation, which are not readily available in most wards. As a result, patients with HTG-AP were commonly admitted to ICU for implementation of plasmapheresis and discharged when a satisfactory triglyceride level was achieved. Thus the indication for ICU admission was not based on disease severity or organ failure but because of the logistics of delivering plasmapheresis. ICU admission is always associated with increased costs, and there is a risk of ICU-related complications, including delirium, anxiety, depression, and posttraumatic stress disorder.^47^ Moreover, plasmapheresis is reported to be associated with multiple potential vascular complications, including catheter-related complications (such as skin rash, pipeline congestion, deep vein thrombosis, perforation, and air embolism), electrolyte disorders, anticoagulation-related bleeding, infection, and allergic reactions.^48^

This study provided evidence that plasmapheresis may not be used in the management of HTG-AP because of the cost and potential complications and because it may not confer any clinical benefit. Of note, patients received different types of plasmapheresis, and the timing was also different, which might impact the results. Definitive and confirmatory evidence would require a randomized controlled trial.

### Strengths and Limitations

This study has several strengths. First, it was based on the largest multicenter cohort study regarding triglyceride-lowering therapy in HTG-AP of which we are aware, and the data were prospectively collected. Second, this study selected OFFD as the primary outcome, and organ failure is the key determinant of AP severity and outcome. Third, we performed PSM and IPTW analyses to reduce patient selection bias and provide a valid comparison between the plasmapheresis and medical therapy.

There are also some limitations. As in all observational studies, confounders were inevitable despite the statistical effort we made. Therefore the clinical implication of our study should be interpreted with caution. Moreover, the cohort involved a fairly small number of patients undergoing plasmapheresis, and the drawback of PSM led to 9 unmatched patients, which further reduced the sample size. Third, HTG-AP only accounts for 4% to 6% of acute pancreatitis cases outside China,^49^ which may impact the generalizability of the results to other countries. Additionally, 69 patients were recruited from the first site (23% of the study participants), suggesting potential center effects, which means hospital-level characteristics might impact the results we observed. Overall, a large randomized trial is needed before a clear recommendation can be made.

## Conclusion

This large, multicenter prospective cohort study of HTG-AP found that early plasmapheresis was not associated with the incidence and duration of organ failure but with a greater need for ICU admission. A definitive randomized controlled trial can be justified in light of these findings.

## Notes

Supplement 1. **eTable 1. **Plasmapheresis Procedure **eTable 2. **Outcomes of Plasmapheresis and Conventional Groups After IPTW **eFigure 1. **Standardized Mean Difference (SMD) of Variables Before and After Propensity Score Matching and Weighting **eFigure 2. **Daily CRP Level in Matched Cohort **eFigure 3. **Daily SOFA Score in Matched Cohort

Supplement 2.
Nonauthor Collaborators

Supplement 3.
Data Sharing Statement
