# Supplementary material for: Early Plasmapheresis Among Patients With Hypertriglyceridemia–Associated Acute Pancreatitis
Source: JAMA Netw Open. 2023 Jun 28;6(6):e2320802. doi: 10.1001/jamanetworkopen.2023.20802 (PMC10308255; doi:10.1001/jamanetworkopen.2023.20802)
Supplement: Supplement 1. — eTable 1. Plasmapheresis Procedure eTable 2. Outcomes of Plasmapheresis and Conventional Groups After IPTW eFigure 1. Standardized Mean Difference (SMD) of Variables Before and After Propensity Score Matching and Weighting eFigure 2. Daily CRP Level in Matched Cohort eFigure 3. Daily SOFA Score in Matched Cohort [file jamanetwopen-e2320802-s001.pdf]

# Supplemental Online Content

Cao L, Chen Y, Liu S, et al; Chinese Acute Pancreatitis Clinical Trials Group (CAPCTG). Early plasmapheresis among patients with hypertriglyceridemia-associated acute pancreatitis. *JAMA Netw Open*. 2023;6(6):e2320802.
doi:10.1001/jamanetworkopen.2023.20802

**eTable 1.** Plasmapheresis Procedure
**eTable 2.** Outcomes of Plasmapheresis and Conventional Groups After IPTW
**eFigure 1.** Standardized Mean Difference (SMD) of Variables Before and After Propensity Score Matching and Weighting
**eFigure 2.** Daily CRP Level in Matched Cohort
**eFigure 3.** Daily SOFA Score in Matched Cohort

This supplemental material has been provided by the authors to give readers additional information about their work.

© 2023 Cao L et al. *JAMA Network Open.*

## eTable 1. Plasmapheresis Procedure

| Characteristic of plasmapheresis procedure | |
|---|---|
| Type | |
| TPE | 50(89) |
| DFPP | 6(11) |
| Initial time | |
| Day1 | 40(71) |
| Day2 | 13(23) |
| After day2 | 3(5) |
| Times | 1(1,1) |
| Plasma Volume, (ml) | 2000(2000,2700) |
| Time/Procedure, (h) | 2.5(2.0,3.0) |

Abbreviations: TPE, therapeutic plasma exhchange; DFPP, double filtration plasmapheresis; IQR, interquartile ranges Continuous normally distributed data were presented as mean (SDs). Skewed continuous data were presented as medians (IQRs). Categorical data were presented as n (%).

© 2023 Cao L et al. *JAMA Network Open.*

## eTable 2. Outcomes of Plasmapheresis and Conventional Groups After IPTW.

| Characteristic | Plasmapheresis | Conventional | P |
|---|---|---|---|
| *Primary outcome* | | | |
| OFFD to day14, median(IQR) | 12.5 (9.0, 14.0) | 13.0 (10.0, 14.0) | .19 |
| *Secondary outcomes* | | | |
| New-onset OF to day14, (n, %) | 120(42.4) | 106 (34.2) | .35 |
| $SOFA_{RANK}$, median(IQR) | -11.0 (-25.0, 0.0) | -5.9 (-23.1, 2.0) | .25 |
| $\Delta SOFA_{max}$, median(IQR) | 1.0 (0.0, 2.0) | 1.0 (0.0, 3.0) | .97 |
| ICU-free days to day14, median(IQR) | 10.0 (7.0, 11.0) | 14.0 (7.0, 14.0) | <.001 |
| Hospital-free days to day 60, median(IQR) | 47.0 (42.2, 52.0) | 51.0 (46.0, 53.0) | .09 |
| ICU need, (n, %) | 264 (93.7) | 112 (36) | <.001 |
| 60-Day Mortality, (n, %) | 10 (3.4) | 7 (2.2) | .55 |
| IPN, (n, %) | 22 (7.9) | 11(3.6) | .16 |

Abbreviations:IPTW, inverse probability of treatment weighting; PE, plasmapheresis; IQR, interquartile ranges; OFFD, organ failure-free day; OF, organ failure; SOFA, Sequential Organ Failure Assessment; ICU, intensive care unit; IPN, infected pancreatic necrosis

Continuous normally distributed data were presented as mean (SDs). Skewed continuous data were presented as medians (IQRs). Categorical data were presented as n (%).

© 2023 Cao L et al. *JAMA Network Open.*

**eFigure 1. Standardized Mean Difference (SMD) of Variables Before and After Propensity Score Matching and Weighting**

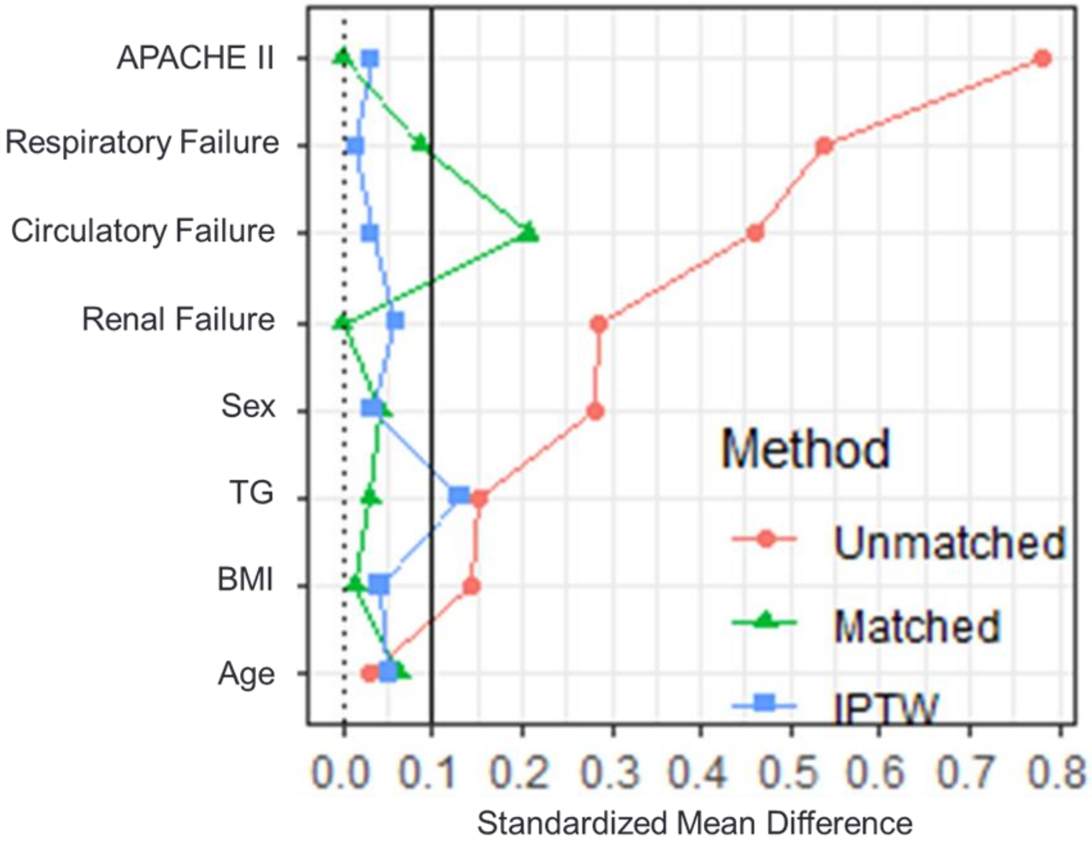


© 2023 Cao L et al. *JAMA Network Open.*

**eFigure 2. Daily CRP Level in Matched Cohort**

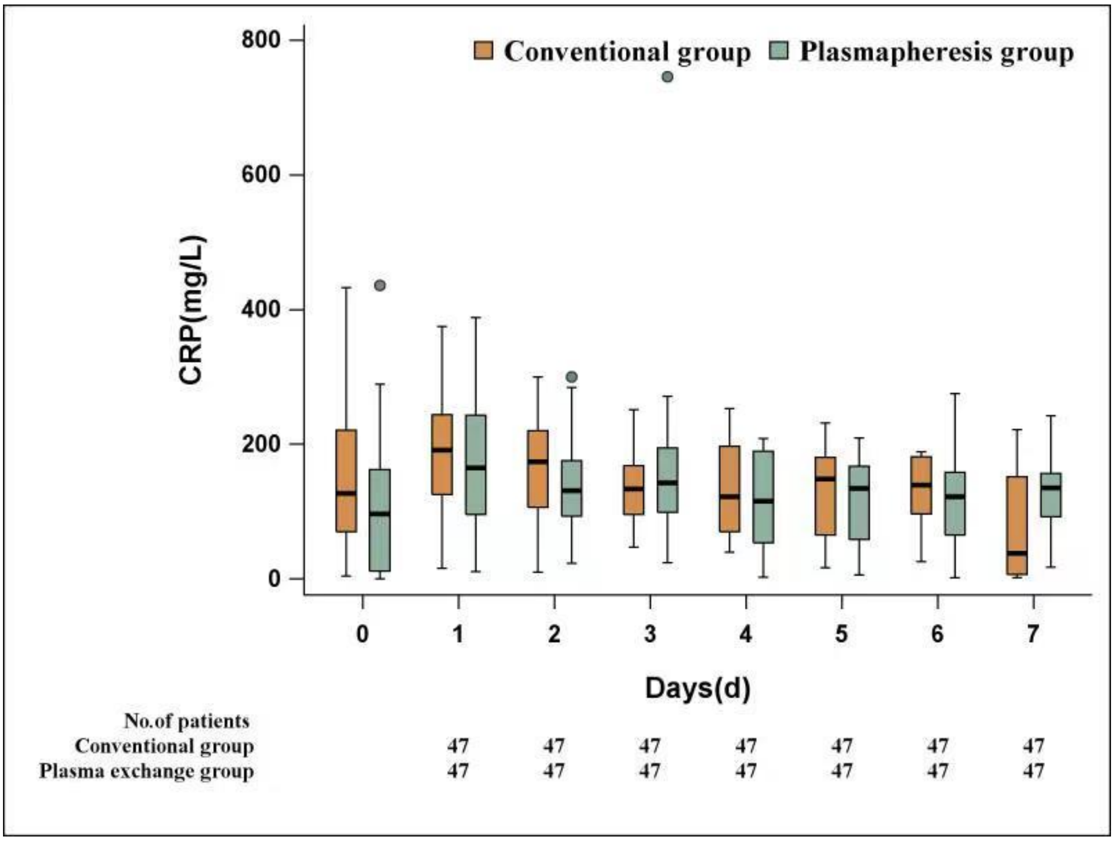


CRP denotes C-reactive protein; IQR denotes interquartile range.

Boxes represent medians and interquartile ranges, whiskers extend to the lowest and highest observations within 1.5 × interquartile range (IQR), and the circles represent outlier observation

© 2023 Cao L et al. *JAMA Network Open.*

## eFigure 3. Daily SOFA Score in Matched Cohort

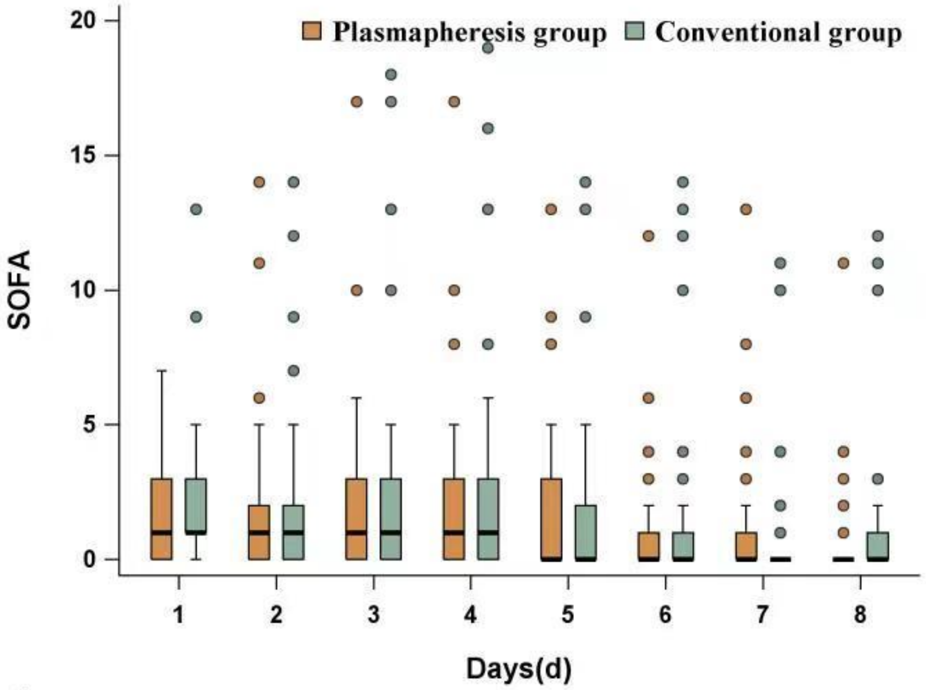


| No.of patients | | | | | | | | |
|---|---|---|---|---|---|---|---|---|
| Conventional group | 47 | 47 | 47 | 47 | 47 | 47 | 47 | 47 |
| Plasma exchange group | 47 | 47 | 47 | 47 | 47 | 47 | 47 | 47 |

SOFA denotes sequential organ failure assessment; IQR denotes interquartile range.

Boxes represent medians and interquartile ranges, whiskers extend to the lowest and highest observations within 1.5 ×IQR, and the circles represent outlier observation.

© 2023 Cao L et al. *JAMA Network Open.*
