# Supplementary material for: Early Plasmapheresis Among Patients With Hypertriglyceridemia–Associated Acute Pancreatitis
Source: JAMA Netw Open. 2023 Jun 28;6(6):e2320802. doi: 10.1001/jamanetworkopen.2023.20802 (PMC10308255; doi:10.1001/jamanetworkopen.2023.20802)
Supplement: Supplement 2. — Nonauthor Collaborators [file jamanetwopen-e2320802-s002.pdf]

Supplemental Online Content: Nonauthor Collaborators
*First name, last name, and suffix (if applicable) are required and will appear in PubMed.

*Group Name(s): the Chinese Acute Pancreatitis Clinical Trials Group (CAPCTG)							
***First Name and Middle Initial(s)**	***Last Name**	***Suffix (eg, Jr, III)**	Academic Degrees	Institution	Location (city, state/province, country)	Role or Contribution, eg, chair, principal investigator	Group (if more than 1 Group listed in the byline) and/or Subgroup (eg, Steering Committee)
Jingchun	Song		PhD	94th Hospital of PLA	Nanchang, Jiangxi, China		
Qingbo	Zeng		PhD	94th Hospital of PLA	Nanchang, Jiangxi, China		
Weili	Gu		PhD	Affiliated Hospital 2 of Nantong University	Nantong, Jiangsu, China		
Weiwei	Chen		PhD	Clinical Medical College of Yangzhou University	Yangzhou, Jiangsu, China		
Qingcheng	Xu		MSc	Clinical Medical College of Yangzhou University	Yangzhou, Jiangsu, China		
Guobing	Chen		PhD	First People's Hospital of Yunnan Province	Kunming, Yunnan, China		
Yafei	Li		MSc	First People's Hospital of Yunnan Province	Kunming, Yunnan, China		
Lijuan	Zhao		PhD	First People's Hospital of Yunnan Province	Kunming, Yunnan, China		
Songjing	Shi		BSc	Fujian Province Hospital	Fuzhou, Fujian, China		
Weijie	Yao		PhD	General Hospital of Ningxia Medical University	Yinchuan, Ningxia, China		
Xiaofei	Huang		PhD	Jiangsu Provincial Hospital of Integrated Chinese and Western Medicin	Nanjing, Jiangsu, China		
Haibin	Ni		PhD	Jiangsu Provincial Hospital of Integrated Chinese and Western Medicin	Nanjing, Jiangsu, China		
Dandan	Zhou		PhD	Jiangsu Provincial Hospital of Integrated Chinese and Western Medicin	Nanjing, Jiangsu, China		
Mingzhi	Chen		BSc	Jinjiang Hospital of Traditional Chinese Medicine	Quanzhou, Fujian, China		
Yan	Chen		BSc	Jinling Hospital, Medical School of Nanjing University	Nanjing, Jiangsu, China		

Supplemental Online Content: Nonauthor Collaborators
*First name, last name, and suffix (if applicable) are required and will appear in PubMed.

***First Name and Middle Initial(s)**	***Last Name**	***Suffix (eg, Jr, III)**	Academic Degrees	Institution	Location (city, state/province, country)	Role or Contribution, eg, chair, principal investigator	Group (if more than 1 Group listed in the byline) and/or Subgroup (eg, Steering Committee)
Mingfeng	Huang		MSc	Jinling Hospital, Medical School of Nanjing University	Nanjing, Jiangsu, China		
Baiqiang	Li		PhD	Jinling Hospital, Medical School of Nanjing University	Nanjing, Jiangsu, China		
Jiajia	Lin		PhD	Jinling Hospital, Medical School of Nanjing University	Nanjing, Jiangsu, China		
Mengjie	Lu		MSc	Jinling Hospital, Medical School of Nanjing University	Nanjing, Jiangsu, China		
Junli	Sun		PhD	Luoyang Central Hospital	Luoyang, Henan, China		
Keke	Xin		MSc	Luoyang Central Hospital	Luoyang, Henan, China		
Chengjian	He		PhD	Nanhua Hospital	Hengyang, Hunan, China		
Hongyi	Yao		PhD	Nanhua Hospital	Hengyang, Hunan, China		
Zigui	Zhu		MSc	Nanhua Hospital	Hengyang, Hunan, China		
Fang	Shao		PhD	Nanjing Medical University	Nanjing, Jiangsu, China		
Yun	Zhou		BSc	Pingxiang People's Hospital	Pingxiang, Jiangxi, China		
Guixian	Luo		BSc	Qianxinan People's Hospital	Xingyi, Guizhou, China		
Xiaomei	Chen		PhD	Qilu Hospital of Shandong University	Jinan, Shandong, China		
Xiangyang	Zhao		PhD	Qilu Hospital of Shandong University	Jinan, Shandong, China		
Wei	Zhao		MSc	Qilu Hospital of Shandong University	Jinan, Shandong, China		
Long	Fu		PhD	Shangqiu First People's Hospital	Shangqiu, Henan, China		
Shumin	Tu		PhD	Shangqiu First People's Hospital	Shangqiu, Henan, China		
Bing	Xue		MSc	Shangqiu First People's Hospital	Shangqiu, Henan, China		
Yongjun	Lin		MSc	Sir Run Run Shaw Hospital of Zhejiang University	Hangzhou, Zhejiang, China		
Xinting	Pan		PhD	The Affiliated Hospital of Qingdao University	Qingdao, Shandong, China		
Youdong	Wan		PhD	The Affiliated Hospital of Qingdao University	Qingdao, Shandong, China		

*First name, last name, and suffix (if applicable) are required and will appear in PubMed.

***First Name and Middle Initial(s)**	***Last Name**	***Suffix (eg, Jr, III)**	Academic Degrees	Institution	Location (city, state/province, country)	Role or Contribution, eg, chair, principal investigator	Group (if more than 1 Group listed in the byline) and/or Subgroup (eg, Steering Committee)
Qingyun	Zhu		PhD	The Affiliated Hospital of Qingdao University	Qingdao, Shandong, China		
Miao	Chen		PhD	The Affiliated Hospital of Zunyi Medical University	Zunyi, Guizhou, China		
Kang	Li		PhD	The Affiliated Hospital of Zunyi Medical University	Zunyi, Guizhou, China		
Hong	Mei		PhD	The Affiliated Hospital of Zunyi Medical University	Zunyi, Guizhou, China		
Dahuan	Li		PhD	The First Affiliated Hospital and College of Clinical Medicine of Henan University of Science and Technology	Luoyang, Henan, China		
Lening	Ren		MSc	The First Affiliated Hospital and College of Clinical Medicine of Henan University of Science and Technology	Luoyang, Henan, China		
Guoxiu	Zhang		PhD	The First Affiliated Hospital and College of Clinical Medicine of Henan University of Science and Technology	Luoyang, Henan, China		
Min	Shao		PhD	The First Affiliated Hospital of Anhui Medical University	Hefei, Anhui, China		
Dongsheng	Zhao		MSc	The First Affiliated Hospital of Anhui Medical University	Hefei, Anhui, China		
Zhenping	Chen		BSc	The First Affiliated Hospital of Nanchang University	Nanchang, Jiangxi, China		
Wenhua	He		PhD	The First Affiliated Hospital of Nanchang University	Nanchang, Jiangxi, China		
Nonghua	Lv		PhD	The First Affiliated Hospital of Nanchang University	Nanchang, Jiangxi, China		
Liang	Xia		PhD	The First Affiliated Hospital of Nanchang University	Nanchang, Jiangxi, China		

Supplemental Online Content: Nonauthor Collaborators
*First name, last name, and suffix (if applicable) are required and will appear in PubMed.

***First Name and Middle Initial(s)**	***Last Name**	***Suffix (eg, Jr, III)**	Academic Degrees	Institution	Location (city, state/province, country)	Role or Contribution, eg, chair, principal investigator	Group (if more than 1 Group listed in the byline) and/or Subgroup (eg, Steering Committee)
Yin	Zhu		PhD	The First Affiliated Hospital of Nanchang University	Nanchang, Jiangxi, China		
Qiang	Li		PhD	The First Affiliated Hospital of Nanjing Medical University	Nanjing, Jiangsu, China		
Honghai	Xia		PhD	The First Affiliated Hospital of the University of Science and Technology of China	Hefei, Anhui, China		
Dongliang	Yang		PhD	The First Affiliated Hospital of the University of Science and Technology of China	Hefei, Anhui, China		
Shusheng	Zhou		PhD	The First Affiliated Hospital of the University of Science and Technology of China	Hefei, Anhui, China		
Weihua	Lu		PhD	The First Affiliated Hospital of Wannan Medical College	Wuhu, Anhui, China		
Jingyi	Wu		PhD	The First Affiliated Hospital of Wannan Medical College	Wuhu, Anhui, China		
Feng	Zhou		MSc	The First Affiliated Hospital of Wannan Medical College	Wuhu, Anhui, China		
Jiyan	Lin		PhD	The First Affiliated Hospital of Xiamen University	Xiamen, Fujian, China		
Quanxing	Feng		PhD	The Fourth Military Medical University	Xian, Shanxi, China		
Mei	Yang		PhD	The Qujing NO1 People’s Hospital	Qujing, Yunnan, China		
Hong	Gao		MSc	The Qujing NO1 People’s Hospital	Qujing, Yunnan, China		
Shan	Xu		MSc	The Second Affiliated Hospital of Chongqing Medical University	Chongqing, China		
Lei	Yu		PhD	The Second Affiliated Hospital of Chongqing Medical University	Chongqing, China		
Bin	Wu		PhD	The Third Hospital of Xiamen City	Xiamen, Fujian, China		
Huaguang	Ye		MSc	The Third Hospital of Xiamen City	Xiamen, Fujian, China		
Zhiyong	Liu		PhD	Xiangya Hospital	Changsha, Hunan, China		

Supplemental Online Content: Nonauthor Collaborators
*First name, last name, and suffix (if applicable) are required and will appear in PubMed.

***First Name and Middle Initial(s)**	***Last Name**	***Suffix (eg, Jr, III)**	Academic Degrees	Institution	Location (city, state/province, country)	Role or Contribution, eg, chair, principal investigator	Group (if more than 1 Group listed in the byline) and/or Subgroup (eg, Steering Committee)
Jianfeng	Tu		PhD	Zhejiang Provincial People's Hospital	Hangzhou, Zhejiang, China		
Hongguo	Yang		MSc	Zhejiang Provincial People's Hospital	Hangzhou, Zhejiang, China		
