# Supplementary material for: Early Plasmapheresis Among Patients With Hypertriglyceridemia–Associated Acute Pancreatitis
Source: JAMA Netw Open. 2023 Jun 28;6(6):e2320802. doi: 10.1001/jamanetworkopen.2023.20802 (PMC10308255; doi:10.1001/jamanetworkopen.2023.20802)
Supplement: Supplement 3. — Data Sharing Statement [file jamanetwopen-e2320802-s003.pdf]

Data Sharing Statement

Cao. Early Plasmapheresis Among Patients With Hypertriglyceridemia–Associated Acute Pancreatitis. *JAMA Netw Open*. Published June 28, 2023.
doi:10.1001/jamanetworkopen.2023.20802

Data
Data available: No

Additional Information
Explanation for why data not available: Deidentified individual participant data are available indefinitely in the electronic database. Data can be accessed through capctg.medbit.cn with the approval of the authors. Request for data can be made to the corresponding author (ctgchina@medbit.cn) and will be discussed during a meeting of the Chinese Acute Pancreatitis Clinical Trials Group (CAPCTG).
